# Decoding cuproptosis and cuproplasia: implications for therapeutic strategies in renal cell carcinoma

**DOI:** 10.1038/s41420-025-02734-z

**Published:** 2025-11-06

**Authors:** Subeka Abraham Gnanadass, Somnath Pandey, Pragasam Viswanathan

**Affiliations:** 1 412-B, Renal Research Laboratory, Pearl Research Park, School of Bio-Sciences and Technology, Vellore Institute of Technology, Vellore, India; 2 Department of Surgery, Sylvester Comprehensive Cancer Center, University of Miami, Miami, FL USA

**Keywords:** Apoptosis, Oncogenesis

## Abstract

Renal cell carcinoma (RCC) is one of the primary drivers of cancer-related mortality worldwide. Despite advancements in cancer diagnosis and management, there is a lack of effective available treatment options for such patients. This is often attributed to late diagnosis, lack of biomarkers, and resistance against standard-of-care therapies. Thus, identifying novel targets in RCC is needed to improve disease outcomes. Cu is a trace element required for homeostasis, and its dysregulation is linked to cancer. Cuproptosis, a programmed cell death, occurs due to intracellular Cu overload, disrupting the TCA cycle, inducing oxidative stress, and impairing metabolism. In cancer, abnormal Cu levels drive Cu-dependent proliferation, termed cuproplasia. The role of cuproptosis and cuproplasia in RCC remains unclear; their comprehensive understanding will enable the discovery of novel targets for effective therapy. This review explores their molecular mechanisms, impact on RCC progression, and therapeutic potential.

## Facts

- Copper (Cu) is an indispensable metal essential for normal biological processes like cellular respiration and iron metabolism, immune function, neurotransmission and cell proliferation. Any dysregulation in Cu levels has been associated with complications like cancer development and progression.
- Cuproptosis is a Cu-induced cell death process initiated by intracellular Cu overload, causing oxidative stress, protein lipoylation, TCA cycle imbalance, and ultimately cell death.
- Dysregulation of Cu levels can induce Cu dependent cancer cell growth, called cuproplasia, which has not been well investigated in RCC.
- Elucidation of the interaction between cuproptosis and cuproplasia in RCC may offer new avenues for targeting copper metabolism for novel therapies.

## Introduction

Renal cell carcinoma (RCC) is a prevalent and lethal genitourinary cancer [1]. A total of 25% of RCC patients have local relapse and metastasis [2]. Moreover, tumor cell malignancy, aberrant metastasis, and proliferation have led to therapeutic resistance [3]. Surgical excision of the lesion remains the primary course of treatment [4]. Though the majority of patients with localized disease still find nephrectomy to be beneficial, studies show that between 20 and 40% of patients with clinically localized RCC will see their cancer return after nephrectomy [5]. Patients with cancer will have a far better prognosis if they are diagnosed in the early stages [6]. Thus, a highly specific target must be identified to effectively combat RCC to provide precision therapy and increase the survival rate.

**Fig. 1:**
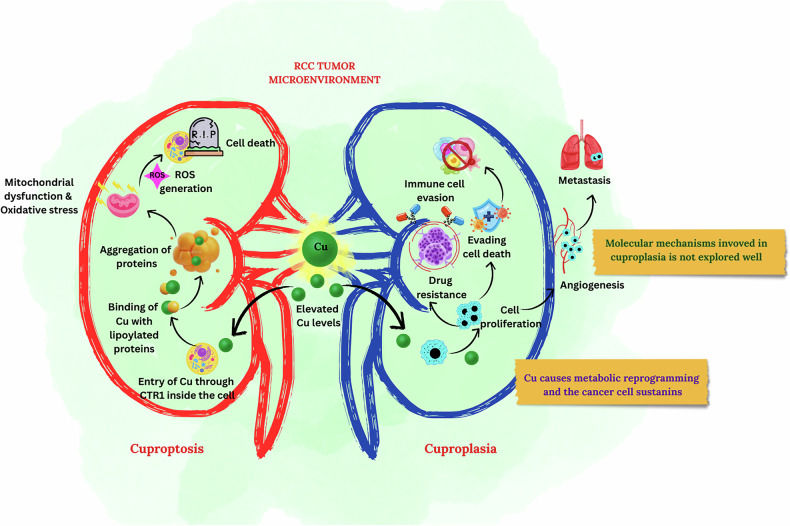
Dysregulation of Cu in RCC microenvironment. This figure summarizes the events that take place when Cu levels are elevated in a cancer environment.

Copper (Cu) is an essential micronutrient required for development in eukaryotes [7]. Because Cu cannot be created or destroyed by metabolic processes, it must be acquired from external sources. Copper exists in 2 oxidation states, Cu^+^ and Cu^2+^. By harnessing the ability of Cu to cycle between two oxidation states, cuproenzymes participate in a wide spectrum of metabolic processes, including aerobic respiration, pigmentation, peptide amidation, catecholamine biosynthesis, iron transport, superoxide dismutation, and biosynthesis of the extracellular matrix. Cuproplasia is a Cu-associated process in which Cu promotes cell growth and proliferation, such as neoplasia, metaplasia, and hyperplasia. Conversely, cuproptosis is a form of programmed cell death initiated by the mitochondrial pathway due to excess Cu. This excess Cu generates proteotoxic stress that ultimately leads to cell death [8]. Recent empirical studies suggest that Cu has a potential role in initiating cancer [9]. Elevated levels of Cu are observed in the serum of cancer patients [10]. These escalated Cu levels may facilitate cancer cell proliferation, angiogenesis, and metastasis [11]. The overview of dysregulated copper in the cancer microenvironment is depicted in Fig. 1.

Recent studies highlight the link between Cu imbalance and cancer, making Cu-targeted therapies promising. Cu-based treatments inducing cell death and inhibiting cuproplasia offer potential interventions. However, the role of Cu in cuproptosis and cuproplasia in RCC remains unclear. This review explores these mechanisms and their therapeutic potential to improve patient outcomes.

## The Cu within us

Cu typically has two biological effects: it is a structural element of proteases and serves as an essential catalytic cofactor in redox processes [12]. It is essential for several biological functions, such as neuropeptide production, free radical detoxification, cellular respiration, cell proliferation, and angiogenesis [13]. Excess Cu in the system can facilitate Fenton-type redox mechanisms, leading to oxidative stress and cell death [14]. Recent studies have illustrated that acute exposure to supraphysiological Cu levels exerts toxic effects, inducing cuproptosis [8]. However, with prolonged exposure, cells adapt and develop resistance to Cu toxicity, resulting in cuproplasia, a Cu-driven proliferative response [15].

Thus, organisms have evolved intricate systems to transport, distribute, and store Cu to reduce its toxicity and maintain its homeostasis.

### Cu metabolism

Inside the body, Cu has two oxidative forms, monovalent Cu ions (Cu+) and divalent Cu ions (Cu^2+^), with its biological functions heavily reliant on the dynamic interplay between the change of ion valence states [16]. Cu is found in the brain, liver, and muscles [17]. Meat and shells are highly rich in Cu [18]. The dietary Cu is directly absorbed by the duodenum and the small intestine [19]. The absorbed Cu binds to ceruloplasmin and albumin for transport to the liver, then is excreted *via* bile into the gastrointestinal tract and eliminated in feces [20].

**Fig. 2:**
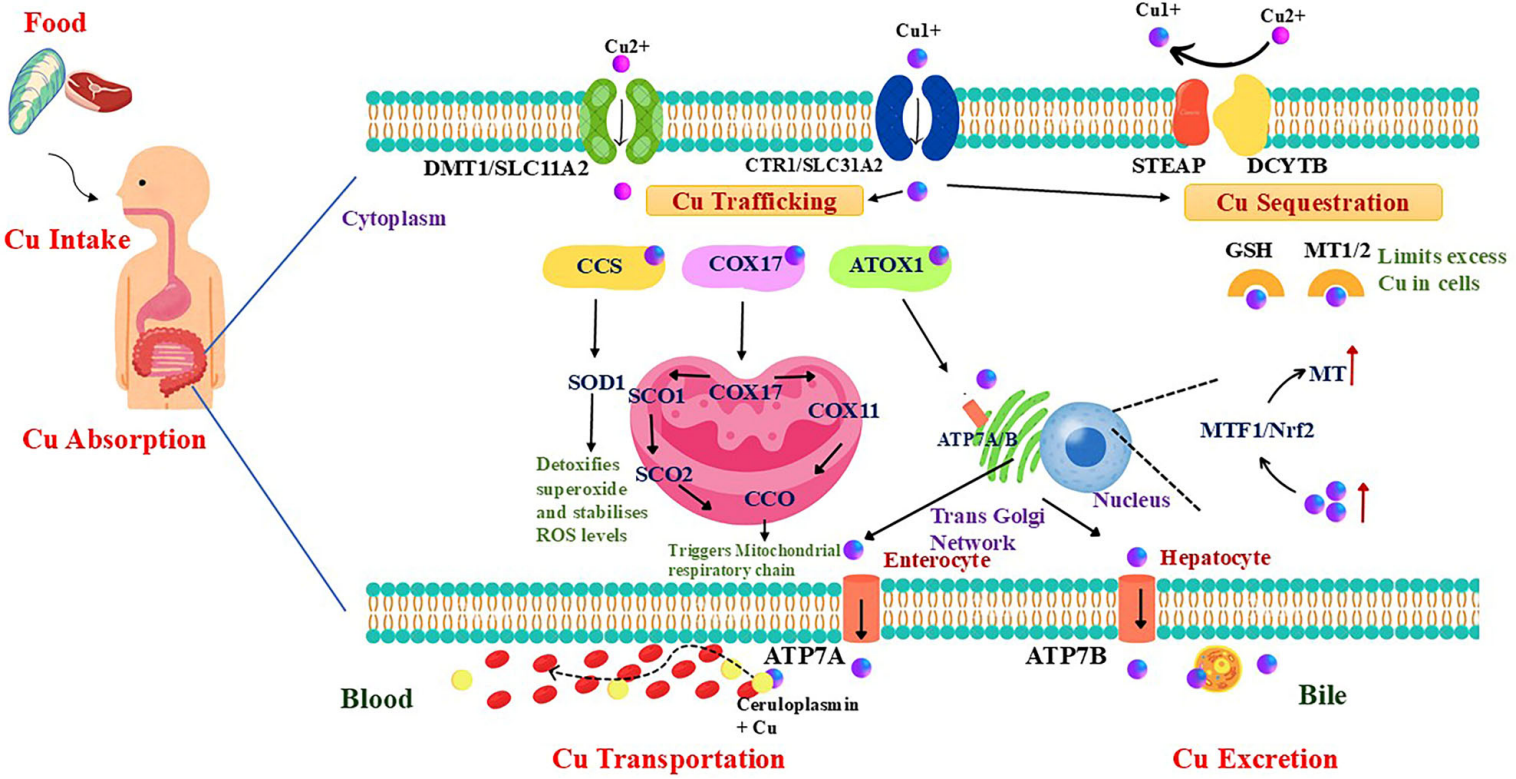
Cu metabolism in the human body. This diagram illustrates copper (Cu) metabolism, from dietary intake to excretion. Ingested Cu is absorbed by enterocytes, with STEAP and DCYTB helping its reduction and transport. Uptake proteins like DMT1 and CTR1 facilitate cytoplasmic entry, where Cu is distributed by CCS, COX17, and ATOX1, that help regulate ROS levels and maintain mitochondrial function. Cu supports enzymes like SOD1 and CCO in detoxification and respiration. Cu homeostasis is maintained by GSH and MT1/2. MT1/2 protein levels are regulated by MTF1/Nrf2 inside cells. Finally, ATP7A/B transports Cu across membranes, binding to ceruloplasmin for blood transport and bile excretion. DMT1 Divalent Metal Transporter 1, SLC11A2 Solute Carrier Family 11 Member 2, CTR1 Copper Transporter 1, SLC31A1 Solute Carrier Family 31 Member 1, STEAP Six-Transmembrane Epithelial Antigen of the Prostate, DCYTB Duodenal Cytochrome, CCS Copper Chaperone for Superoxide Dismutase, COX17 Cytochrome C Oxidase Copper Chaperone COX17, ATOX1 Antioxidant 1 Copper Chaperone, GSH Glutathione, MT1/2 Metallothionein 1 and 2, SOD1 Superoxide Dismutase 1, SCO1 Synthesis of Cytochrome C Oxidase 1, SCO2 Synthesis of Cytochrome C Oxidase 2, COX17 Cytochrome C Oxidase Copper Chaperone 17, COX11 Cytochrome C Oxidase Assembly Protein 11, CCO Cytochrome C Oxidase, ATP7A/B ATPase Copper Transporting Alpha/Beta.

The absorption of Cu in the small intestine is carried out by Cu transporter 1 (CTR1) through the enterocyte plasma membrane [19]. The plasma membrane has metalloreductase proteins like duodenal cytochrome b (DCYTB) and six-transmembrane epithelial antigen of prostate (STEAP) which facilitate the reduction of Cu^2+^ ions to Cu^+^ ions and transport them into the cells via CTR1 [21], whereas Cu^2+^ ions can be directly absorbed by the divalent metal transporter 1 (DMT1) protein [22]. Cu trafficking depends on a group of proteins, ensuring that Cu gets delivered to its precise location to maintain the levels of Cu in our body. Figure 2 shows CCS, ATOX1, and COX17, implicated in Cu distribution. Cu²⁺ reduction to Cu⁺ transfers electrons to oxygen, generating ROS. To prevent ROS accumulation, Cu chaperone for superoxide dismutase (CCS) facilitates transport of Cu to SOD1, detoxifying superoxides and stabilizing ROS levels [23]. Also, Cu chaperone antioxidant 1 (ATOX1) delivers Cu to Cu ATPase proteins like ATPase Cu-transporting alpha (ATP7A) and ATPase Cu-transporting beta (ATP7B). It transports Cu within the trans-golgi network and other cellular compartments [24]. Cu binds with cytochrome oxidase (COX), an enzyme in electron transport, and presents it to the synthesis of cytochrome c oxidase 1 and 2 (SCO1 and SCO2) and participates in the redox pathway and mitochondrial respiratory chain. Also, Cu is transported to the cytochrome c oxidase (COX) subunits via COX 17 and COX 11 and produces ATP [25].

Cu sequestration is another process that takes place to control the overload of Cu inside the cell. The excess Cu in the cells is bound to Glutathione (GSH) and Metallothionein (MT1/2) [26]. Also, the Cu overload triggers the activity of transcription factors of metal regulatory transcription factor 1 (MTF1) and Nuclear respiratory factor 1 (NRF1), and produces more MT proteins to limit the access of excess Cu [27]. Then the Cu in enterocytes is transported to the blood by ATP7A, and in hepatocytes is transported by ATP7B to the bile [26]. The overview of Cu transport and metabolism is depicted in Fig. 2.

## Dysregulation of Cu

Cu homeostasis is crucial for maintaining various physiological processes. Dysregulation of Cu in humans can lead to significant health issues across various organ systems, particularly affecting the lungs, liver, and central nervous system [25].

### Cuproplasia

Cuproplasia, the abnormal Cu-dependent growth and proliferation of tumor cells, significantly influences cancer progression and patient outcomes [15]. Complex signaling cascades regulate it and can be involved in Cu’s enzymatic and non-enzymatic roles. The term “cuproplasia” delineates the role of Cu in promoting cellular proliferation and growth, particularly in the context of hyperplasia, metaplasia, and neoplastic transformations [15]. It is also associated with cellular mechanisms like mitochondrial respiration, antioxidant defense, autophagy, redox, and kinase signaling [26, 28, 29]. A recent paper explored Cu-induced mechanisms driving RCC progression through multiomic approaches, including metabolomics, lipidomics, metallomics, and transcriptomics, using various cell lines, xenograft models, and patient tumors [30]. Bischoff et al. showed how ccRCC adapts its metabolism as it accumulates copper during cancer development and progression. In advanced or recurring tumors, copper levels were higher and more copper was directed to a key energy-producing enzyme called cytochrome C oxidase. When researchers limited copper in the diet of mice with tumors, tumor growth slowed or stopped, but giving cancer cells extra copper in the lab activated several growth and energy-making pathways. Copper enhanced the cells’ ability to use oxygen in their mitochondria, promoted the assembly of large energy complexes, and boosted the pentose phosphate pathway, which provides building blocks for DNA and helps handle harmful molecules. Copper also supported the making of new DNA building blocks and influenced how the cells used glucose both for energy and for making glutathione (GSH), a molecule that protects from damage. When these copper-managed processes were disrupted, the cancer cells could not keep a healthy balance, leading to a buildup of hydrogen peroxide and, because of copper’s toxicity, cell death.

The authors further investigated deep-sequencing analysis, and their single-cell RNA sequencing (scRNA-seq) and spatial transcriptomics revealed coordinated upregulation of respiratory complex subunits and genes related to GSH and Cu metabolism during ccRCC progression. These findings by Bischoff et al. highlight Cu-driven metabolic reprogramming through mitochondrial CuCOX activation, oxidative stress detoxification, and enhanced cancer cell proliferation, ultimately driving ccRCC progression [30].

The abnormality of cuproplasia is controlled by Cu-selective chelators and metal ionophores [31]. Moreover, cuproplasia can be modulated by pharmacological or genetic manipulation of proteins involved in Cu homeostasis [15]. Further research is needed to know the molecular mechanisms behind the initiation and progression of cuproplasia and its role in pathological conditions.

### Cuproptosis

While Cu is an essential trace mineral for various biological functions, excessive Cu intake can lead to serious health consequences. Although several cell death processes, including pyroptosis, necroptosis, apoptosis, and ferroptosis, have been thoroughly studied, it is still unknown how exactly Cu ions cause cellular toxicity and cell death [32]. Tsvetkov and his team discovered a unique Cu-dependent programmed cell death process called cuproptosis. They used Cu ionophore to investigate the role of Cu in cell death mechanisms. Cu ionophores are tiny ion carrier molecules that shuttle Cu into the cells. Tsvetkov et al. suggest that elevated Cu levels are the primary cause of cell death when exposed to the Cu ionophore elesclomol (ES). The results showed that the cleavage or activation of caspase 3, a sign of apoptosis, was not involved in ES-induced cell death [33]. Cu-induced cell death is distinct from apoptosis because neither the deletion of the essential apoptotic effectors, BAX and BAK1, nor the co-treatment of the cells with pan-caspase inhibitors affected ES-induced cell death. Similarly, ES-induced cell death was unaffected by pharmacological therapy with inhibitors of ferroptosis or necroptosis [8].

Tsvetkov et al. also proposed that Cu-associated toxicity induces cuproptosis through mitochondrial respiration and metabolism [8]. The authors discovered that cells that relied on mitochondrial respiration were considerably more vulnerable to ES than those that relied on glycolysis, suggesting that mitochondrial activity had a major influence on the susceptibility to Cu ionophores. Furthermore, treatment with inhibitors that target the mitochondrial pyruvate uptake and electron transport chain (ETC) significantly decreased the susceptibility to Cu dependent cell death, confirming the role of mitochondrial respiration in cuproptosis. In contrast, treatment with the mitochondrial uncoupling agent dissipates the proton gradient and reduces ATP synthesis without blocking electron transport, indicating that mitochondrial respiration is primarily responsible for Cu-induced cell death rather than ATP synthesis. The authors also state that ES does not reduce the basal or ATP-related respiration, but dramatically reduces the respiratory reserve capacity- the extra mitochondrial capacity available to respond to increased energy demand. This indicates that Cu did not directly target the ETC but rather the impaired TCA cycle components. Further, highlighting the significance of cellular respiration in Cu-induced cell death, they demonstrated that cell culture under hypoxic conditions significantly reduces the sensitivity to cell death compared to normoxic conditions. A significant reduction in the susceptibility to Cu dependent cell death was also observed in hypoxic cell culture compared to normoxic cell culture, highlighting the significance of cellular respiration in Cu-induced cell death [8]. Therefore, more research is needed to determine the exact mechanisms behind the connection between the TCA cycle and Cu-induced cell death.

Tsvetkov et al. also link lipoylation to cuproptosis. Lipoylation is a highly preserved post-transcriptional modification where lipoamide is covalently bonded to a lysine residue via an amide bond. This process is known to only happen on four crucial multimeric metabolic complexes in mammals, which control particular carbon entry points into the TCA cycle’s primary metabolic route [34]. Tsvetkov et al. pinpointed the precise metabolic pathways implicated in Cu toxicity by the unexpected reduction in the amount of cell death caused by Cu ionophores when the following genes were silenced, it was established that FDX1, an iron-sulfur protein that is a direct target of ES and can convert Cu^2+^ into its more hazardous form Cu^1+^, is an upstream regulator of protein lipolylation. Moreover, dihydrolipoamide S-succinyltransferase (DLST), a different TCA cycle component, and DLAT’s protein lipoylation both significantly decreased in FDX1 knockdown. These proteins showed impaired Cu-binding ability, indicating the requirement of the lipoyl moiety for Cu binding. Additionally, a model was put out that suggested that the aberrant oligomerization of lipoylated proteins—specifically, DLAT was responsible for the harmful gain-of-function that occurred after exposure to Cu ionophores [8]. The connection was further emphasized by mass spectrometric analysis, which demonstrated that Cu ionophore treatment resulted in iron-sulfur cluster protein loss and consequent proteotoxic stress in an FDX1-dependent manner [8].

Cuproptosis is involved in the generation of reactive oxygen species (ROS) [8]. The relationship between oxidative stress and cuproptosis was discovered by studying in vitro and in vivo models of intervertebral disc degeneration. Oxidative stress promoted cuproptosis in the presence of Cu by upregulating FDX1 and TCA-related proteins, and it boosted Cu influx by elevating the expression of CTR1 and ATP7A [35]. This work demonstrated the significance of oxidative stress as a mediator between the induction of cuproptosis and elevated Cu levels [8]. Similarly, quantitative proteomics demonstrated the interaction between cuproptosis and Cu stress in cancer cells [36]. It was shown that Cu stress caused cuproptosis, oxidative damage, ROS overproduction, and cell cycle arrest. Positive cuproptosis mediators were down-regulated and negative ones were up-regulated in response to Cu treatment. This suggests that cuproptosis in cancer functions as a feedback mechanism to prevent excessive Cu absorption. However, sufficient use of Cu led to the inevitable demise of cancer cells through cuproptosis [8].

Recent investigations reveal that there exists a strong relationship between the metal-dependent cell death, like cuproptosis, and mitochondrial quality control (MQC) pathways involved in regulation of mitochondrial integrity and turnover, such as mitochondrial dynamics and mitophagy. The MQC pathways modulate cellular sensitivity to Cu-induced proteotoxic stress. Although MQC functions to keep mitochondrial function intact and block excessive ROS production, its dysregulation can either inhibit or enhance cuproptosis based on the cellular context. This intricate interaction highlights the role of mitochondria in deciding the destiny of cancer cells treated with copper overload [37].

### Other complications

**Fig. 3:**
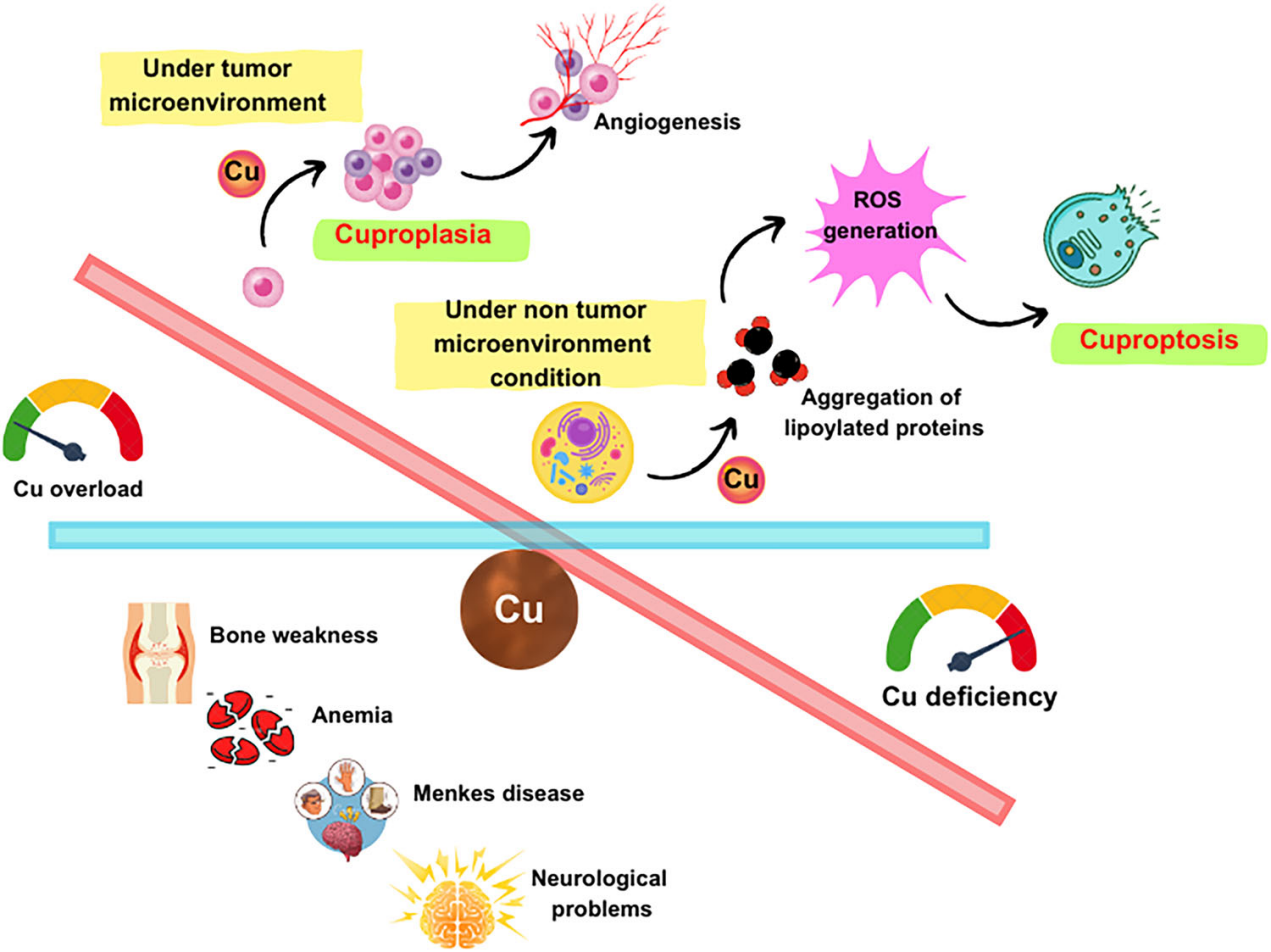
Dysregulation of Cu in the human body. The figure highlights Cu’s dual role in physiology and pathophysiological states like cancer and copper-related disorders. As an essential cofactor, Cu supports enzyme function and balance. Excess Cu drives cuproplasia, fueling tumor growth, while in non-tumor conditions, cuproptosis—triggered by lipoylated protein aggregation and ROS—offers a potential therapy. Conversely, Cu deficiency is linked to neurological issues, bone weakness, anemia, and Menkes disease.

Dysregulation of Cu leads to genetic diseases. Wilson’s disease is a genetic disorder resulting from a defective ATP7B gene that prevents the body from processing Cu. This leads to the accumulation of excess Cu in both the liver and the brain, leading to serious health consequences. Serious symptoms include liver damage, which can lead to liver failure and neurological problems such as difficulty moving and changes in mood or behavior [38]. Deficiency of Cu leads to Menke’s disease. This is characterized by the mutation of the Cu ATPase protein ATP7A, which helps in the transport of Cu from enterocytes to other regions [39]. Further deficiency of Cu leads to bone weakness and anemia [40, 41]. Studies also suggest that deficiency in Cu leads to other complications like Non-alcoholic fatty liver disease, Obesity, Parkinson’s disease, Alzheimer’s disease, and Diabetes Mellitus [42–44]. The overview of the Cu dysregulation is depicted in Fig. 3.

## The Cu-cancer link

Cu is tightly linked to the development of cancer as it binds to important molecules involved in tumor-associated signaling pathways potentially triggering the progression of cancer either directly or indirectly. Dysregulation of Cu metabolism is highly associated with the progression of cancer. Cu overload is observed in a variety of malignancies and it has a great impact on proliferation, angiogenesis, and metastasis [13].

Higher levels of Cu are often found in cancer tissues and the serum of cancer patients, including breast, gynecological, lung, pancreatic, gastric, esophageal, colorectal, prostate, brain, gallbladder, and blood cancers [15]. Higher Cu concentrations guarantee higher energy requirements for the rapid division of cancer cells, as Cu is a crucial cofactor of COX in mitochondria [9]. Raised Cu levels affect not only mitochondrial function and energy production but also proliferation, angiogenesis, glycolysis, lipid metabolism, and extracellular matrix. It has been shown that these processes are essential for the proliferation, angiogenesis, metastasis, and chemoresistance of cancer cells [27].

In the context of cuproplasia, Cu overload triggers neoplasia and hyperplasia and alters the Cu-regulating genes or the attachment of Cu to the regulatory sites of proteins. Cu activates important cancer signaling pathways like 3-phosphoinositide-dependent protein kinase 1 (PDK1), phosphoinositide-3-kinase/Akt pathway, and epidermal growth factor receptor (EGFR) pathway [9]. For example, Cu binds with the tyrosine kinase (RTK) and activates it without the requirement of a ligand. This further activates the agammaglobulinemia tyrosine kinase (AKT) and extracellular regulated protein kinase (ERK), resulting in cell proliferation and metastasis. It also binds to histidine 203 and 117 regions of PDK1 and activates the AKT pathway [45]. This further enhances the distribution of forkhead box O1a (FoxO1a) and forkhead box O4 (FoxO4) and triggers cancer development and metastasis [46]. Cu can directly bind to mitogen-activated protein kinase kinase 1 (MEK1) to promote the phosphorylation of ERK1/2 and further activate the downstream c-Jun N-terminal kinase (JNK) to regulate tumor growth [47]. Cu ions promote the shedding of the notch ligand Jagged1 on the cell surface and promote tumor cell migration [48].

Numerous reports have revealed that Cu ions have a close relationship with tumor angiogenesis through enhancing the pro-angiogenic factors like angiopoietins (Ang), vascular endothelial growth factor (VEGF), fibroblast growth factors (FGFs), placental growth factor (PLGF), interleukins (IL), and integrins [27]. Cu triggers angiogenesis, mainly stabilizing the hypoxia-inducible factor 1 subunit alpha (HIF-1α), which further promotes the activity of proangiogenic factors and cancer [49].

Emerging evidences indicate that Cu actively regulates epithelial-mesenchymal transition (EMT), a key mechanism in cancer progression and metastasis. For instance, lysyl Oxidase Like (LOXLs) and lysyl oxidase (LOX) play a major role in ECM remodeling by catalyzing the amino group deamination in elastin and collagen, leading to cross-linking and further increasing the matrix [50]. Elevated LOX levels have been shown to enhance the invasion of cancer cells in breast cancer [51]. Similarly, exposure of lung cancer cells to CuSO_4_ has been found to enhance the expression of LOXL2, promoting fibrogenic changes [52]. These findings strongly imply the association of Cu in triggering cancer. Thus, a deeper understanding of this mechanism might underpin new avenues for cancer treatments.

Recent investigations suggest that Cu is involved in cuproptosis resistance mechanisms. For instance, Cu influences the Wnt/β-catenin signaling pathway and upregulates ATP7B transporter, which further impairs the intercellular Cu accumulation and helps the cancer cells to survive even under Cu overload [53]. As a whole, Cu plays a significant role in triggering energy metabolism, angiogenesis, evading cell death mechanisms, and metastasis, which provides evidence that Cu triggers cancer progression and paves a way for Cu associated treatments based on the copper cancer biology.

### Role of Cu dysregulation in renal cell carcinoma

#### Genes associated with Cu dysregulation in RCC

Dysregulation of Cu-associated genes is widely observed in many cancers. Pan cancer analysis of CRGs at epigenetic, genetic, and transcriptional levels provides insights into cancer’s CRG levels and prognosis. It also suggests that it is involved in the mTOR pathway and that using mTOR inhibitors would clinically benefit ccRCC patients [54]. A recent report showed that gene clustering for cuproptosis could be used to predict survival and therapeutic responses in patients with ccRCC. Moreover, inhibiting cuproptosis pathways may provide new therapeutic possibilities, especially through modulating essential regulators like MTF1 [55]. The down-regulation of cuproptosis regulator MTF1 triggers the progression of kidney renal clear cell carcinoma (KIRC) and positively correlates with immune infiltration in the RCC environment [56]. Also, CRG, like CDKNA2, acts as a great prognostic factor related to KIRC, and it is highly associated with the immune mechanisms that pave the way for KIRC immunotherapy [57]. Recent studies have identified novel cuproptosis-related genes in predicting the prognosis of ccRCC [58]. CDKNA2 is also involved in predicting overall survival in RCC. Higher expression of CDKNA2 tends to worsen the OS of ccRCC [59]. DLAT is frequently downregulated in cancers. In ccRCC, the gene and protein expression of DLAT is downregulated and associated with poor survival. Also, it is strongly associated with tumor immune infiltration [60]. Also, DLAT overexpression suppresses the migration of ccRCC cells by inducing cuproptosis [61]. Similarly, FDX1 acts as a tumor suppressor role in ccRCC, in silico approaches implies that it can be a great prognostic marker and highly correlate with immune infiltration in ccRCC [62]. Also, in ccRCC, the tumor tissues showed a marked reduction in FDX1 expression level. The expression patterns are highly associated with clinicopathological features and prognosis. Further, it leads to a shorter survival period and higher immune cell activation [63]. In another study, FDX1 positively influenced oxidative phosphorylation and fatty acid metabolism. Also, the overexpression of FDX1 in ccRCC cell lines suppressed tumor growth and strengthened the immune response against the tumor by enhancing the secretion of the TNFγ and IL-2 [64]. FDX1 also plays a crucial role in drug resistance mechanisms. Adrenomedullin overexpression activates p38/MAPK signaling mechanisms and promotes the phosphorylation of FOXO3 and makes a way to access the nucleus. In the nucleus, FOXO3 inhibits the FDX1 transcription and causes sunitinib drug resistance in ccRCC [65]. Recently, the downregulation of FDX1 and DLAT in ccRCC leads to poor survival and is highly associated with immune checkpoints and immune infiltration [66]. Contradictory to that, the knockdown of FDX1 and PDHB reduces the proliferation and migration of ccRCC cells [67]. Similarly, PDHB is highly associated with ccRCC progression. Low levels of PDHB are an unfavorable indicator in survival analysis. Further, in advanced malignancies, the expression of PDHB is relatively low, leading to higher tumor progression. The findings of Che et al. identified that RHBDL2 interacts with the cuproptosis regulators. The knockdown of RHBDL2 triggers the activity of FDX1 and blocks the migration and proliferation of ccRCC cells. Also, abnormal expression of RHBDL2 might suppress the immune system and trigger immune escape [68]. DLST is another potential cuproptosis regulator that is downregulated in ccRCC and is involved in cancer progression through immune-associated mechanisms [69].

A study by Li H et al. explored the immunological, clinical, and pharmacological roles of cuproptosis regulators in ccRCC [60]. A study by Wang et al. found that cuproptosis is linked to immune infiltration, with patients grouped into three clusters: C1 (low cuproptosis), C2 (moderate), and C3 (high). C1 patients respond well to anti-PDL1 therapy, while C3 patients show significant changes in immune-regulating genes, such as amplifying immune checkpoint proteins like PDL-1, CTLA-4, PD-1 and deleting immunomodulators [70]. The summarized research findings of cuproptosis-related mRNAs are depicted in Table 1.

**Table 1:** Role of mRNA associated with Cu dysregulation in cancer.

mRNA	Expression	Role	Reference
MTF1	Downregulated	Triggers RCC cell proliferation and regulates RCC immune microenvironment	[56]
CDKNA2	Upregulated	Activates CD4/8+ T and worsens the prognosis of KIRC	[57]
		Helps to predict overall survival in ccRCC	[59]
DLAT	Downregulated	Lower gene and protein expression in ccRCC leads to poor survival and is involved in stronger tumor immune infiltration.	[60]
		Overexpression of DLAT induces cuproptosis and suppresses the proliferation and migration of ccRCC cells	[61]
FDX1	Downregulated	Regulates fatty acid metabolism and oxidative phosphorylation. Overexpression of FDX1 strengthens the immune system against tumor by triggering the secretion of TNFγ and IL-2	[64]
		FDX1 involved in mitochondrial dysfunction and acts as a better prognostic marker for ccRCC	[120]
		Predicts prognosis in ccRCC and it is highly associated with immune infiltration	[62]
		Adrenomedullin inhibits FDX1 expression and makes ccRCC cells resistant to sunitinib	[65]
		miR-21-5p is the upstream regulator of FDX1 and inhibiting miR-21-5p has a great therapeutic value.	[85]
		Strongly associated with prognosis and clinicopathological features of ccRCC. Also has a shorter survival period and tumor immune activation	[63]
FDX1 DLAT	Downregulated	This leads to poor survival and is closely associated with immune checkpoints and immune infiltration.	[66]
FDX1 PDHB	Downregulated	Knockdown of the CRGs might reduce the proliferation and migration of ccRCC.	[67]
RHBDL2	Upregulated	The knockdown of RHBDL2 triggers FDX1 expression and blocks the proliferation and migration of ccRCC cells.	[68]
DLST	Downregulated	Initiates cancer progression through immune-associated mechanisms	[69]

Together, these observations make CRGs novel diagnostic and prognostic biomarkers and highlight their potential for combination therapy targets, which incorporate copper modulation, agents that target metabolism, and immunotherapy. Nonetheless, to fully exploit the clinical potential of cuproptosis, further translational work will be needed to dissect the exact molecular pathways, optimize treatment windows, and adequately stratify patients.

#### ncRNAs associated with Cu dysregulation in RCC

A significant percentage of the human genome (76–97%) consists of RNA transcripts that do not make proteins, known as non-coding RNAs (ncRNAs) [71]. Since their discovery, scientists have increasingly recognized the crucial roles of ncRNAs in various biological processes. This has led to a paradigm shift in our understanding of RNA, from merely a messenger for protein production to a multifaceted molecule actively involved in gene regulation and the organization of genetic material. They also play a crucial role in transcriptional regulation, epigenetic, and post-transcriptional regulation [72]. ncRNAs comprise both small and long ncRNAs. The major types of small ncRNAs include micro-RNA (mi-RNA) and circular RNA (circRNA). Even though these ncRNAs don’t make proteins, they still significantly impact various physiological processes through the control of their target genes. Dysregulation of ncRNAs is frequently observed in cancers, as it can be both oncogenic and tumor suppressor [73].

Emerging studies have shown that ncRNAs can influence cuproptosis and cuproplasia in cancer [74]. Many prognostic models have been based on ncRNAs in cancer. Zhang et al. identified prognostic lnc RNAs (AC091212.1, FOXD2-AS1, AC026401.3, LINC00460, and C007365.1), which can be associated with immune checkpoints and might direct immune checkpoint blockade immunotherapy [75]. Also, in another study lncRNAs (LINC02154, SUCLG2-AS1,NUP153-AS1,FOXD2-AS1 and LINC00271) helps to predict prognosis in ccRCC [76].

Similarly, Bai et al. imply that lnc RNAs (LASTR, FOXD2-AS1, and AC026401.3) predict prognosis and immune response in ccRCC patients [77]. Also, in mccRCC patients, MINCR, FOXD2-AS1, and LINC02154 are upregulated and predict the patient response to ICI-based therapy [78]. Similarly, FOXD2-AS1, AC091057.1, LINC00839, and AP003119.3 have significant correlations with immune response and the sensitivity of tumor cells to various chemotherapeutic drugs [79]. Studies also suggest that AC004837.2, FOXD2-AS1, MINCR, and LINC02154 are highly expressed and lead to poor survival. Also, LINC01671 has a protective role in RCC, but it is downregulated, and it needs more experimental validation to know its role [80]. In another study cuproptosis-related lncs (PICSAR, LINC02154, H1-10-AS1, LINC02027, HHLA3, LINC00471, SNHG15, EIF1B-AS1, SNHG8, and MINCR) have great potential in the diagnosis of ccRCC and it is associated with immune mechanisms in ccRCC [81]. Recent machine learning approaches identified cuproptosis and disulfidptosis-related lnc RNAs (MANEA-DT, AC095055.1, ACVR2B-AS1, and AL161782.1) as potential predictors of prognosis in ccRCC. However, experimental validation implies that ACVR2B-AS1 has a protective role in prognosis prediction, while MANEA-DT is involved in poor prognosis [82].

The above findings suggest that ncRNAs have great potential in regulating cuproptosis and cuproplasia with important implications for prognosis, immune response, and therapeutic responsiveness in RCC. Their frequent co-occurrence with immune checkpoints and response to therapy not only makes them ideal biomarkers but also strengthens their role for a combinational therapeutic strategy. For instance, LINC02154 is upregulated in ccRCC and makes the immune environment highly active. Additionally, knockdown of LINC02154 increases the expression of cuproptosis regulators, such as FDX1 and DLAT, and inhibits the cell proliferation and migration of ccRCC cells [83]. Similarly, LINC01711 is highly expressed in KIRC cells; the knockdown of LINC01711 blocks the migration and invasion of KIRC cells [84]. Recent investigations suggest that microRNAs are involved in regulating the cuproptosis process. For instance,miR-21-5p is significantly upregulated in ccRCC. This miRNA post-transcriptionally modulates the expression of FDX1 and initiates the ccRCC progression [85]. Further research on experimental verification and mechanistic understanding, focusing on miRNAs and circRNAs, is needed to fully exploit their potential in precision oncology for RCC. The summarized research findings of cuproptosis-related ncRNAs are depicted in Table 2.

**Table 2:** Role of nc RNA associated with Cu dysregulation in cancer.

nc-RNA	Type of ncRNA	Role	Reference
AC091212.1 FOXD2AS1 AC026401.3 LINC00460 C007365.1	Long non-coding RNA	It is involved in predicting the RCC prognosis and it is associated with immune checkpoint regulation.	[75]
AC026401.3 FOXD2-AS1 LASTR	Long non-coding RNA	It predicts prognosis and immune response in ccRCC patients	[77]
MINCR FOXD2-AS1 LINC02154	Long non-coding RNA	Upregulated in mccRCC patient’s blood who receive ICI treatment and involved in the prediction of the patient’s response in ICI-based therapy	[78]
FOXD2-AS1 AC091057.1 LINC00839 AP003119.3	Long non-coding RNA	Highly correlated with immune response and chemotherapeutic drugs	[79]
AC004837.2 FOXD2-AS1 MINCR LINC02154	Long non-coding RNA	Overexpressed in renal cells and leads to poor survival	[80]
LINC01671	Long non-coding RNA	It has a protective role, but needs further experimental validation to know its role	[80]
PICSAR LINC02154 H1-10-AS1 LINC02027 HHLA3 LINC00471 SNHG15 EIF1B-AS1 SNHG8 MINCR	Long non-coding RNA	It has higher diagnostic efficiency and is significantly associated with the immune status of ccRCC patients.	[81]
MANEA-DT AC095055.1 ACVR2B-AS1 AL161782.1	Long non-coding RNA	ACVR2B-AS1 has a protective role and can be a great prognostic marker. In contrast, MANEA-DT involved in poor prognosis	[82]
LINC00271, FOXD2-AS1, NUP153-AS1, SUCLG2-AS1, LINC02154	Long non-coding RNA	Helps to predict patient survival and in the classification of subtypes	[76]
OVCH1-AS1, AC103706.1, SBF-AS1, CDK6-AS1, AC034236.3 LINC02154, AC002451.1,	Long non-coding RNA	They help us to predict prognosis in KIRC patients. It also shows promising responses to immunotherapy, as low-risk patients respond well to immunotherapy	[121]
FDXD2-AS1, AC091212.1, LINC00460, AC026401.3, AC007365.1	Long non-coding RNA	They are associated with poor prognosis and the GSEA analysis showed that the TCA cycle and pyruvate metabolism are involved in low-risk patients.	
LINC02027, GNG12-AS1, SNHG8, LINC01963, CAHM, ZNF32-AS1, BDNF-AS, APPCDD1L-DT	Long non-coding RNA	They are associated with poor prognosis and predict the overall survival of KIRC patients. Further, the potentially effective drugs were also identified to treat KIRC	[85]
LINC02154	Long non-coding RNA	Positively regulates immune cell mechanisms in ccRCC. Also, knockdown of LINC02154 leads to the upregulation of cuproptosis regulators FDX1 and DLAT	[83]
LINC01711	Long non-coding RNA	LINC01711 is upregulated in KIRC cells and knockdown of LINC01711 blocks the migration and invasion of KIRC cells	[84]
NFE4	Long non-coding RNA	m6A associated ncRNA NFE4 was significantly upregulated in ccRCC cells. In the presence of Copper and Elesclomol, the expression of NFE4 was downregulated, but in prolonged exposure, the NFE4 was high, indicating its role in cuproptosis resistance mechanisms	[122]
miR-21-5p	Micro RNA	miR-21-5p post-transcriptionally regulates FDX1 and initiates RCC progression.	[85]

### Cu-based therapeutics for cancer

Recent research suggests that manipulating Cu levels within cancer cells could be a powerful new approach to treating cancer [86]. Cu-based therapeutics are being explored for renal cancer treatment due to Cu’s significant role in tumor growth and metabolism. Cu-based therapies are current trends in cancer as they disrupt the Cu homeostasis in cancer cells and trigger cancer cell death. Cu in cancer can be utilized in therapy either by elevating Cu levels with “Cu ionophores” or diminishing them with “Cu chelators”. While these Cu-based therapeutic strategies show promise in preclinical studies, clinical trial data specific to renal cancer remain limited. Ongoing research and future clinical trials are essential to further elucidate the efficacy and safety of these approaches in renal cancer treatment. Nonetheless, Cu-based therapies that have been employed for other cancer types teach us a lot. The Cu-based cancer therapies are described below.

### Cu Ionophores

Although the recently found cuproptosis is receiving more attention, and it is being well established in Wilson’s disease, in which Cu overload causes liver cell damage. Cu overload can also induce cell death in cancer cells by inducing a toxic accumulation of reactive oxygen species (ROS). Although cancer cells tend to have higher levels of ROS compared to normal cells, they are still susceptible to additional ROS increases induced by excess Cu. Cu ionophores are molecules that binds to the metals and carry them from the extracellular membrane to the intracellular membrane, resulting in the accumulation of metals inside the cell [87]. Further, the accumulation of Cu triggers interacts with the cellular membranes and triggers ROS production and leading to cell death [88]. Thus ionophores helps in triggers cuproptosis in cancer cells.

#### Disulfiram (DSF)

Disulfiram (Antabuse), a drug approved by the FDA for the treatment of alcoholism since 1951, has been used safely in the clinic for many years [89]. It is effective because it inhibits aldehyde dehydrogenase (ALDH), resulting in the accumulation of acetaldehyde when alcohol is ingested, producing unpleasant effects and deterring drinking [90]. DSF is a potential ionophore that has gained attention in cancer therapeutics. DSF has also recently been found to have potential as an anticancer agent in laboratory experiments. In an acidic environment, DSF is hydrolyzed into N, N-diethyldithiocarbamate (DDTC). DDTC subsequently forms a DDTC-Cu^2+^ complex with Cu. The complex exerts a strong anticancer activity via several mechanisms involving inhibition of both NF-κB and TGF-β signaling, apoptosis, PKM2-induced aerobic glycolysis, angiogenesis and ferroptosis [46, 91]. Also, DSF acts as a Cu ionophore by hydrolyzing into DDTC and chelates Cu to form a stable, lipophilic Cu(DDC)_2 complex facilitating the transport of Cu across the plasma membrane without the CTR1 transporter. This Cu catalyzed the redox reactions, producing higher amount of ROS, leading to mitochondrial damage and cell death [91, 92]. Experiments in mice with implanted human glioblastoma cells (U87) demonstrated that the disulfiram-Cu complex can inhibit tumor growth by inhibiting angiogenesis through the inhibition of VEGF [93]. Disulfiram has also been found to inhibit angiogenesis in other cancers such as osteosarcoma, cervical and renal carcinomas, and in some cell types (CL1-5, NTUB1, and HUVEC) by lowering the levels of matrix metalloproteinases MMP-9 and MMP-2 [94]. DSF + Cu inhibits RCC growth and dissemination and enhances outcomes through the induction of increased oxidative stress and induction of ferroptosis. Furthermore, the inhibition of the NRF2 through NPL4 inhibition further augments DSF/Cu-induced oxidative stress and ferroptosis [95].

#### Elesclomol

Elesclomol, a new drug that was originally developed by Synta Pharmaceuticals as an adjunct to chemotherapy, functions by interfering with cancer cells [96]. Elesclomol functions as a Cu ionophore by forming a 1:1 stable complex with Cu extracellularly [97]. Then it shuttles between the exterior and interior membrane of the cell, facilitating the transport of Cu ions into cells. This increases the intracellular Cu ions in cells [98]. At the same time, elesclomol has been shown to degrade copper-transporting ATPase 1 (ATP7A) in colon cancer cells, a protein responsible for intracellular export of copper. This degradation of ATP7A by elesclomol also results in the accumulation of copper ions within the mitochondria of cancer cells [99]. Through its action as a Cu ionophore, it is dependent on Cu to generate toxic ROS that subsequently trigger mitochondrial apoptosis, resulting in cancer cell death [100]. Elesclomol triggers cancer cell death by inducing unbalanced oxidative stress, a Cu-dependent and energy-consuming process [101, 102]. Although certain cancer cells can evade the action of proteasome inhibitors by adopting oxidative phosphorylation, this metabolic adaptation renders them vulnerable to elesclomol alone [48, 103]. A Phase II study of the combination of elesclomol and paclitaxel in metastatic melanoma demonstrated good results with a highly improved progression-free survival [104]. Nevertheless, a larger Phase III trial could not establish overall efficacy in the average population of melanoma. Analysis of this trial revealed that elesclomol can be effective in cancer patients with tumors dependent on mitochondrial metabolism and low levels of LDH [100].

Other than drugs, Cu-associated proteins like membrane proteins, chaperon proteins, and intercellular proteins help carry Cu in the system. Further Cu ionophores also have some minimal off-target effects, which can be rectified using nano-drug delivery systems, designing the Cu ionophores as a protonophore, and chemically modifying the ionophores. Research on this is at an early stage, but underpinning the potential of these proteins might give a new perspective on cancer therapy.

### Cu chelators

Tumor cells thrive in Cu-rich environments [105]. Elevated Cu adds fuel for cancer cells to multiply and metastasize. The only way to eradicate cancer cells is to block the Cu availability to the cancer cells, which makes it an attractive strategy to maintain Cu in a cancer environment. Cu chelators decrease the bioavailability of Cu in cancer cells. Cu chelators are nothing but molecules that bind to Cu and reduce its bioavailability, further suppressing the cell proliferation, angiogenesis, and metastasis in cancer environments [106].

#### Tetrathiomolybdate (TTM)

Tetrathiomolybdate (TTM), a potential Cu chelator, is widely used to treat Wilson disease [107]. Emerging studies imply that TTM effectively inhibits tumor growth by reducing Cu absorption. This can be achieved by inhibiting Cu-dependent mitochondrial complex IV, which reduces mitochondrial respiration and increases oxygen availability. This elevated oxygen level activates HIF-prolyl hydroxylase (PHD), and triggers angiogenesis by degrading HIF-1α. This, in turn, suppresses the expression of pro-angiogenic factors like PDK1, GLUT1, and VEGF [108]. Apart from this Cu chelating effect, TTM also inhibits cell growth induced by mutant KRAS^G12D^ in CRC through a decrease in ATP7A expression [109]. Also, TTM’s anti-angiogenic and anti-tumor effects have been strengthened in animal models, which demonstrated that it reduces tumors by Cu depletion and inhibition of pro-angiogenic factor production through NF-κB [110, 111]. TTM also inhibits tumor development in TTM-treated Her2/neu and FVB mice and induces architectural alterations in breast tissue that decrease blood vessel density, eventually halting tumor development in the breast [112].

Clinical trials have demonstrated that TTM retards tumor progression in early-stage malignant mesothelioma likely due to its anti-angiogenic properties. In an advanced kidney tumors phase II trial, though TTM successfully depleted Cu, it also caused elevated levels of some pro-angiogenic factors like IL-8 and IL-6, indicating a multifactorial interaction between Cu depletion and these factors. In summary, these data imply that TTM’s anti-tumor action is largely due to lowering Cu-dependent enzyme activity, and combining it with other treatments may be advantageous [113].

#### D-Penicillamine (D-Pen)

D-penicillamine, or D-pen, is a drug that has been in use since the 1950s. It functions by eliminating excess Cu from the body. D-pen functions as a mono, di, and tridentate ligand by binding to the donor atoms with the help of the thiol group and stabilizes the Cu ions to Cu chelates [114]. This D-pen-Cu complex is effectively removed via urine, which lowers the Cu accumulation. During chelation, D-pen can reduce both Cu+ and Cu2+, which generates a higher amount of hydrogen peroxide (H_2_O_2_) [115]. Experiments have indicated that D-pen, when combined with Cu, has the potential to form toxic molecules known as reactive oxygen species (ROS). It can also inhibit some proteins that facilitate the growth and proliferation of tumors. Further, it has been found that this combination can suppress the formation of new blood vessels in tumors and kill certain immune cells that support tumors to grow [116, 117]. In the glioblastoma multiforme study, Cu sulfate + DPA reduces the proliferation and invasion of glioblastoma cells with high Cu concentration [118]. Also, DPA + Oxaliplatin suppresses oxaliplatin resistance cervical cancer growth [119]. Chelators have a great role in cancer therapy. More research should be done on utilizing Cu chelators in RCC to explore its potential in RCC therapy. Also, the off-targets of the Cu chelators can be rectified using targeted delivery systems, dose optimization of drugs, and choosing highly selective Cu chelators.

Even though clinical studies have been completed and can offer some possibilities for treating cancer, as detailed in Table 3. Clinical trials of Cu-targeting therapy in other cancers offer possible insights to improve RCC treatment strategies. First, helps to understand the mechanisms through which Cu facilitates tumor growth, metastasis, and other hallmarks of cancer, which might provide the basic molecular landscape of Cu interaction in the tumor environment. Second, phase trials of Cu chelators or other Cu-interfering agents in cancers can provide useful information on efficacy, toxicity, and dosing, which could be followed in RCC. Third, understanding the role of Cu in the sustenance of cancer stem cells or resistance to standard therapy, gleaned from experiments in other cancers, could reveal novel therapeutic targets in RCC, particularly in advanced or refractory diseases. Fourth, biomarker evaluation in these trials, measurement of Cu content or Cu-related protein expression, could identify potential subgroups of RCC patients most likely to benefit from Cu-targeted therapy, allowing more individualized treatment regimens.

**Table 3:** Cu-based therapeutics in clinical trials.

Drug	Cancer	Trial no	Phase	Status
Cu + Disulfiram	Castration-Resistant Prostate Cancer	NCT02963051	Ib	Terminated
Cu + Disulfiram	Metastatic Breast Cancer	NCT03323346	II	Recruiting
Cu 64 PSMA I&T injection	Prostate Cancer	NCT05653856	II	Completed
Cu Isotope	Head and Neck Cancer	NCT02864836	–	Unknown
Disulfiram and Cu gluconate	Solid tumors in the liver	NCT00742911	I	Completed
Disulfiram + Cisplatin	Refractory germ cell tumors	NCT03950830	II	Completed
Disulfiram	Recurrent Glioblastoma	NCT02678975	II/III	Completed
Disulfiram	Advance Gastric Cancer	NCT05667415	–	Not yet recruiting
Disulfiram + Chemotherapy	Metastatic Pancreatic Cancer	NCT02671890	I	Terminated
Disulfiram + Chemotherapy	Lung Cancer	NCT00312819	II/III	Completed
Elesclomol Sodium	Solid Tumors	NCT00827203	I	Suspended
Elesclomol (STA-4783) + Paclitaxel	Melanoma	NCT00522834	III	Terminated
Elesclomol Sodium + Docetaxel and Prednisone	Metastatic Prostate Cancer	NCT00808418	I	Completed
STA-4783 and Paclitaxel	Solid Tumors	NCT00088114	I	Completed
Elesclomol Sodium	Relapsed or Refractory Acute Myeloid Leukemia	NCT01280786	I	Unknown
Elesclomol Sodium and Paclitaxel	Recurrent or Persistent Ovarian Epithelial Cancer, Fallopian Tube Cancer, or Primary Peritoneal Cancer	NCT00888615	II	Completed
STA-4783 in Combination With Weekly Paclitaxel	Soft Tissue Sarcomas	NCT00087997	II	Completed
STA-4783 + Paclitaxel and Carboplatin	Non-Small Cell Lung Cancer	NCT00088088	I/II	Completed
Clioquinol	Relapsed or Refractory Hematologic Malignancy	NCT00963495	I	Terminated
Penicillamine	Recurrent Head and Neck Cancer	NCT06103617	–	Recruiting
Penicillamine	Glioblastoma	NCT00003751	II	Completed
Trientine + Pegylated Liposomal Doxorubicin and Carboplatin	Epithelial Ovarian Cancer	NCT03480750	I/II	Completed
Trientine With Vemurafenib	BRAF Mutated Metastatic Melanoma ClinicalTrials.gov ID NCT02068079	NCT02068079	I	Withdrawn
Trientine and Carboplatin	Advanced Malignancies	NCT01178112	I	Completed
Tetrathiomolybdate	Hormone Refractory Prostate Cancer	NCT00150995	II	Completed
Tetrathiomolybdate	Breast Cancer	NCT00195091	II	Terminated
Tetrathiomolybdate	Hepatocellular Carcinoma	NCT00006332	II	Completed
Tetrathiomolybdate + Carboplatin/Pemetrexed	Metastatic Non-small Cell Lung Cancer	NCT01837329	I	Completed
Tetrathiomolybdate + Capecitabine + Pembrolizumab	Triple Negative Breast Cancer	NCT06134375	Ib/II	Recruiting
Radiation Therapy and Ammonium Tetrathiomolybdate	Non-Small Cell Lung Cancer	NCT00560495	II/III	Withdrawn
Chemoradiation and Tetrathiomolybdate	Esophageal Carcinoma	NCT00176800	II	Completed
ATN-224	Prostate Cancer	NCT00405574	II	Unknown
ATN-224 and Temozolomide	Advanced Melanoma	NCT00383851	II	Unknown
Exemestane With or Without ATN-224	Recurrent or Advanced Breast Cancer	NCT00674557	II	Terminated
ATN-224 and Bortezomib	Multiple Myeloma	NCT00352742	I/II	Terminated

-The information furnished above is obtained from (https://clinicaltrials.gov/).

Additional study is necessary to address the limits of Cu metal-binding compounds for cancer treatment, which are still in the early stages of development. The difficulty of selectively targeting cancer cells is a significant obstacle in this field. Also, currently, research evidence primarily focuses only on the mRNA level. To gain a more comprehensive picture, further studies should focus on non-coding RNA, especially on microRNAs and Circular RNAs.

## Conclusion and future direction

Cu is essential for cellular functions, affecting cancer progression and cell death, and requires tight intracellular homeostasis. Its dysregulation in RCC is an active area of research. Though cuproptosis is a potential therapeutic target and cuproplasia drives RCC, the threshold of the processes induced by Cu accumulation is not conclusively proven. The exact threshold is for these processes to become activated is something that needs to be investigated further. Investigating the molecular switches between these processes could provide further insights into mechanisms controlling cuproptosis and cuproplasia. Machine learning and bioinformatics have brought an enhanced understanding of Cu’s function in cancer, yet experimental confirmation is scarce. A majority of RCC research depends on computational models associating cuproptosis-linked genes with results. Bridging this gap between experiments and predictions is the driving force for developing Cu-targeted therapies.

The role of copper in initiating cuproplasia in RCC is hypothetical and lacks experimental validation. More experimental validation of cuproptosis and cuproplasia in identifying RCC subtypes, association with drug resistance, role in metabolic reprogramming, interaction with tumor immune microenvironment, and association with key genes involved in RCC progression might decipher novel treatment strategies for RCC treatment. Also, Cu-based clinical trials are in their infancy and need much exploration, but the accumulating evidence from preclinical models provides a solid foundation for translational progress. Considering the ambivalent nature of copper as oncogenic and cytotoxic, upcoming RCC trials would be well-advised to look into combination approaches—combining copper modulators with VEGF inhibitors or immunotherapy checkpoint blockade—to leverage the context-dependent nature of copper. As our comprehension of cuproplasia and cuproptosis advances on the molecular front, the addition of copper-directed therapies to RCC clinical trials promises a new and exciting horizon of personalized oncology.
